# *Gli2* gene-environment interactions contribute to the etiological complexity of holoprosencephaly: evidence from a mouse model

**DOI:** 10.1242/dmm.026328

**Published:** 2016-11-01

**Authors:** Galen W. Heyne, Joshua L. Everson, Lydia J. Ansen-Wilson, Cal G. Melberg, Dustin M. Fink, Kia F. Parins, Padydeh Doroodchi, Caden M. Ulschmid, Robert J. Lipinski

**Affiliations:** 1 Department of Comparative Biosciences, School of Veterinary Medicine, University of Wisconsin-Madison, Madison, WI 53706, USA; 2 Molecular and Environmental Toxicology Center, School of Medicine and Public Health, University of Wisconsin-Madison, Madison, WI 53706, USA

**Keywords:** Holoprosencephaly, Birth defects, Gene-environment, Hedgehog signaling

## Abstract

Holoprosencephaly (HPE) is a common and severe human developmental abnormality marked by malformations of the forebrain and face. Although several genetic mutations have been linked to HPE, phenotypic outcomes range dramatically, and most cases cannot be attributed to a specific cause. Gene-environment interaction has been invoked as a premise to explain the etiological complexity of HPE, but identification of interacting factors has been extremely limited. Here, we demonstrate that mutations in *Gli2*, which encodes a Hedgehog pathway transcription factor, can cause or predispose to HPE depending upon gene dosage. On the C57BL/6J background, homozygous GLI2 loss of function results in the characteristic brain and facial features seen in severe human HPE, including midfacial hypoplasia, hypotelorism and medial forebrain deficiency with loss of ventral neurospecification. Although normally indistinguishable from wild-type littermates, we demonstrate that mice with single-allele *Gli2* mutations exhibit increased penetrance and severity of HPE in response to low-dose teratogen exposure. This genetic predisposition is associated with a *Gli2* dosage-dependent attenuation of Hedgehog ligand responsiveness at the cellular level. In addition to revealing a causative role for GLI2 in HPE genesis, these studies demonstrate a mechanism by which normally silent genetic and environmental factors can interact to produce severe outcomes. Taken together, these findings provide a framework for the understanding of the extreme phenotypic variability observed in humans carrying *GLI2* mutations and a paradigm for reducing the incidence of this morbid birth defect.

## Abstract (precis)

**Summary:** This work illustrates how a specific genetic predisposition in combination with exposure to an environmental factor can result in a severe birth defect, providing a new opportunity to develop prevention strategies.

## INTRODUCTION

Holoprosencephaly (HPE) is the most prevalent human forebrain malformation, and one of the most common of all developmental abnormalities, with an estimated prevalence of 1 in 250 conceptuses (Leoncini et al., 2008; Orioli and Castilla, 2010; Matsunaga and Shiota, 1977). HPE is defined by incomplete midline division of the embryonic forebrain and frequently co-occurs with facial abnormalities, including hypotelorism, ophthalmologic anomalies, midfacial hypoplasia and orofacial clefts (Richieri-Costa and Ribeiro, 2010; Cohen, 2006; Pineda-Alvarez et al., 2011). In surviving individuals, HPE causes severe intellectual disability, and learning, behavior and motor impairment (Cohen, 2006). More often, the severity of effects results in prenatal or perinatal mortality (Solomon et al., 2010).

Clinical presentation of HPE is extremely variable. True HPE ranges from alobar forms, marked by a single ventricle and no separation of the cerebral hemispheres, to lobar forms with minor midline deficiencies (Solomon et al., 2010). However, obligate carriers of HPE-associated mutations frequently exhibit facial dysmorphology in the absence of detectable brain abnormalities, or no apparent phenotype (Solomon et al., 2012). The dramatic variability in phenotypic expression is believed to stem from a complex heterogeneous etiology involving the interaction of genetic and environmental factors (Krauss and Hong, 2016). Although this premise has become widely accepted, supportive experimental evidence demonstrating specific interacting factors is limited. This knowledge gap hinders clinical management of HPE by limiting the accuracy of genetic counseling and stymying development of prevention strategies (Mercier et al., 2010).

In humans and animal models, HPE has been linked to chemical and genetic disruption of the Hedgehog (Hh) signaling pathway (Roessler et al., 1996; Chiang et al., 1996; Heyne et al., 2015a). Initiated by the Sonic Hedgehog (SHH) ligand, Hh signaling is required for ventral patterning and expansion of the medial forebrain, as well as outgrowth of the processes that form the midface. Hh-signal transduction culminates in regulation of tissue-specific target genes by the Gli family of zinc finger transcription factors, with GLI2 acting as the dominant transcriptional activator (Lipinski et al., 2006). Serving as a poignant example of the complexity that has frustrated basic and translational research efforts, the role of *GLI2* in HPE has remained unclear for two reasons. First, although single-allele *GLI2* mutations have been detected in individuals with HPE-like phenotypes, the majority of mutation carriers do not exhibit the full manifestation of HPE or are clinically unaffected (Bear et al., 2014; Franca et al., 2010; Roessler et al., 2003; Bertolacini et al., 2012; Rahimov et al., 2006). Second, *Gli2*-knockout mice generated on an outbred CD-1 background do not recapitulate the forebrain and facial abnormalities that characterize human HPE (Matise et al., 1998; Park et al., 2000).

We examined the effect of GLI2 loss of function by backcrossing a null allele to the C57BL/6J (B6) background, which has been shown to exacerbate craniofacial phenotypes, including HPE. We found that a homozygous *Gli2* mutation on the B6 background causes the salient features of severe human HPE, including abnormalities of the face and forebrain. B6 *Gli2^+/−^* mice and cells were then used to test whether normally silent single-allele mutations increase sensitivity to a class of teratogens that includes environmental compounds. These functional *in vivo* and mechanistic *in vitro* assays demonstrated a gene-environment interaction that provides a basis to potentially reduce the incidence of this etiologically complex disease in susceptible human populations.

## RESULTS

### GLI2 loss of function causes HPE

**Fig. 1.**
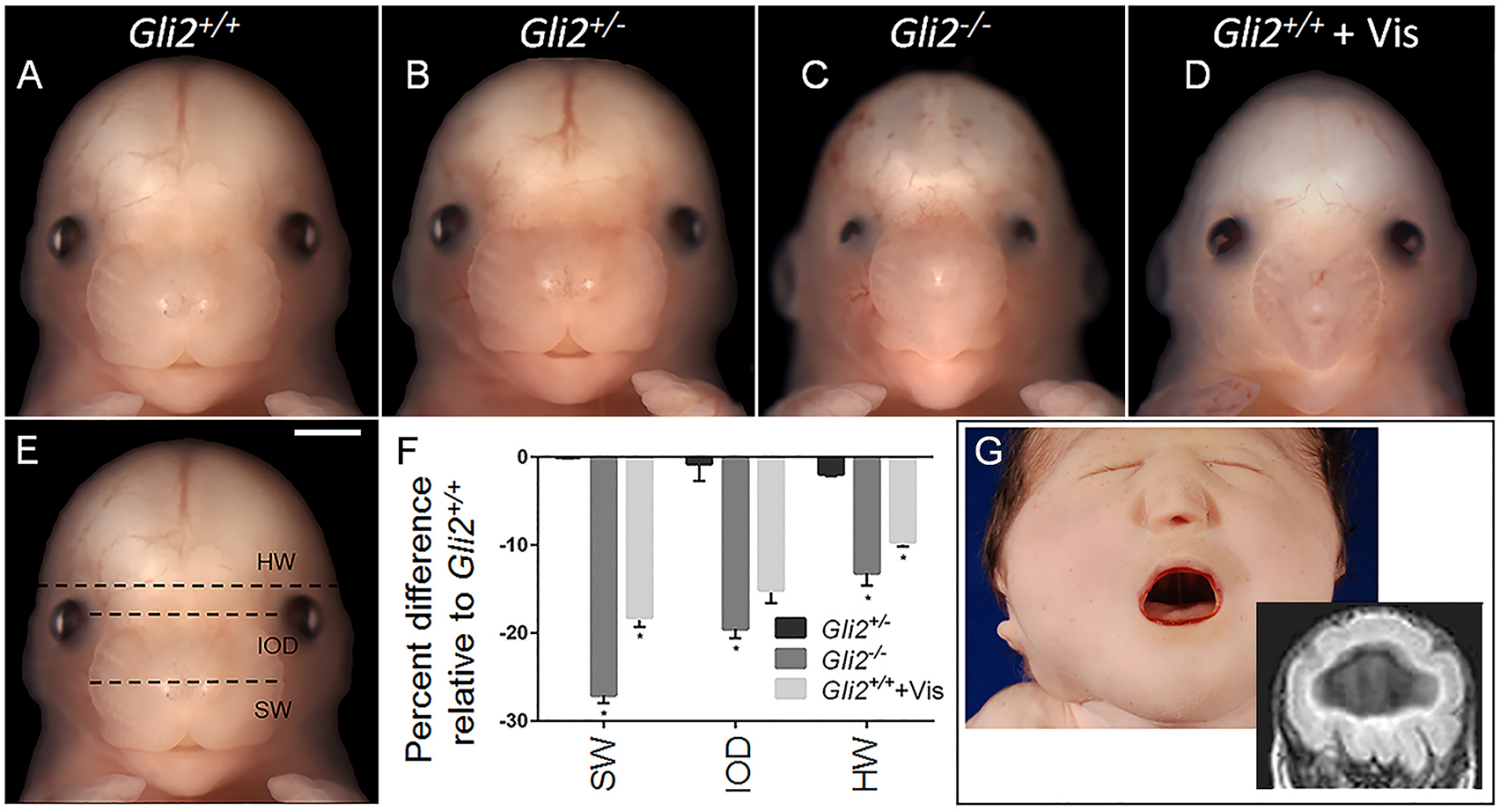
**GLI2 loss of function causes HPE-associated facial dysmorphology.** (A-D) B6 *Gli2^+/+^*, *Gli2^+/−^* and *Gli2*^−/−^ fetuses at GD15 are shown along with a *Gli2^+/+^* fetus that had been exposed to 40 mg kg^−1^ vismodegib (Vis) at GD7.75. Snout width (SW), interocular distance (IOD) and head width (HW) were measured, as illustrated by the dashed lines shown in E. (F) Linear measurements were normalized to *Gli2^+/+^* control group values and are shown on a semi-log plot. Values represent the mean±s.e.m. (*Gli2^+/+^ n*=6; *Gli2^+/−^ n*=9; *Gli2*^−/−^ *n*=6; *Gli2^+/+^*+ Vis *n*=12). *P≤0.05 by one-way ANOVA followed by Tukey's HSD. (G) A neonatal child exhibits the face and brain phenotypes of alobar HPE. A single central nostril, hypotelorism and midfacial hypoplasia co-occur with an undivided forebrain and single ventricle shown by prenatal imaging. Scale bar: 1 mm.

Mating of B6 *Gli2^+/−^* mice generated *Gli2*^−/−^ fetuses at the expected Mendelian ratio (*n*=13 out of 49) at gestational day (GD)15. Midfacial hypoplasia and hypotelorism were observed in all *Gli2^−/−^* animals on the B6 background (Fig. 1). These abnormalities co-occurred with absence of the upper lip notch and a single central or two closely opposed nostrils. The range of facial phenotypes observed in *Gli2^−/−^* fetuses is shown in Fig. S1. Significant reductions in both snout width and interocular distance were identified by taking linear measurements. *Gli2^−/−^* animals also displayed microphthalmia and decreased head width, suggestive of microcephaly. Facial morphology in B6 *Gli2^+/−^* fetuses was indistinguishable from that of wild-type littermates. Crown-rump length and limb morphology were not different between B6 *Gli2^+/+^*, *Gli2^+/−^* and *Gli2^−/−^* fetuses (Fig. S2).

*Gli2^−/−^* animals were also compared to wild-type B6 fetuses that had been exposed to a single 40 mg kg^−1^ of bodyweight dose of the potent Hh-signaling pathway antagonist vismodegib at GD7.75. This teratogenic exposure regimen has recently been reported to result in severe HPE phenotypes (Heyne et al., 2015a). A remarkable degree of overlap in facial dysmorphology was observed among *Gli2^−/−^* fetuses and those acutely exposed to vismodegib (Fig. 1). Importantly, these facial phenotypes in mice closely mimic those observed in humans with severe forms of true HPE. This is illustrated in the neonate shown in Fig. 1G, who exhibits severe midfacial hypoplasia, hypotelorism and a single central nostril. Magnetic resonance imaging (MRI) conducted before birth showed that these facial features co-occurred with HPE, illustrated by an undivided cerebral cortex and single ventricle.

**Fig. 2.**
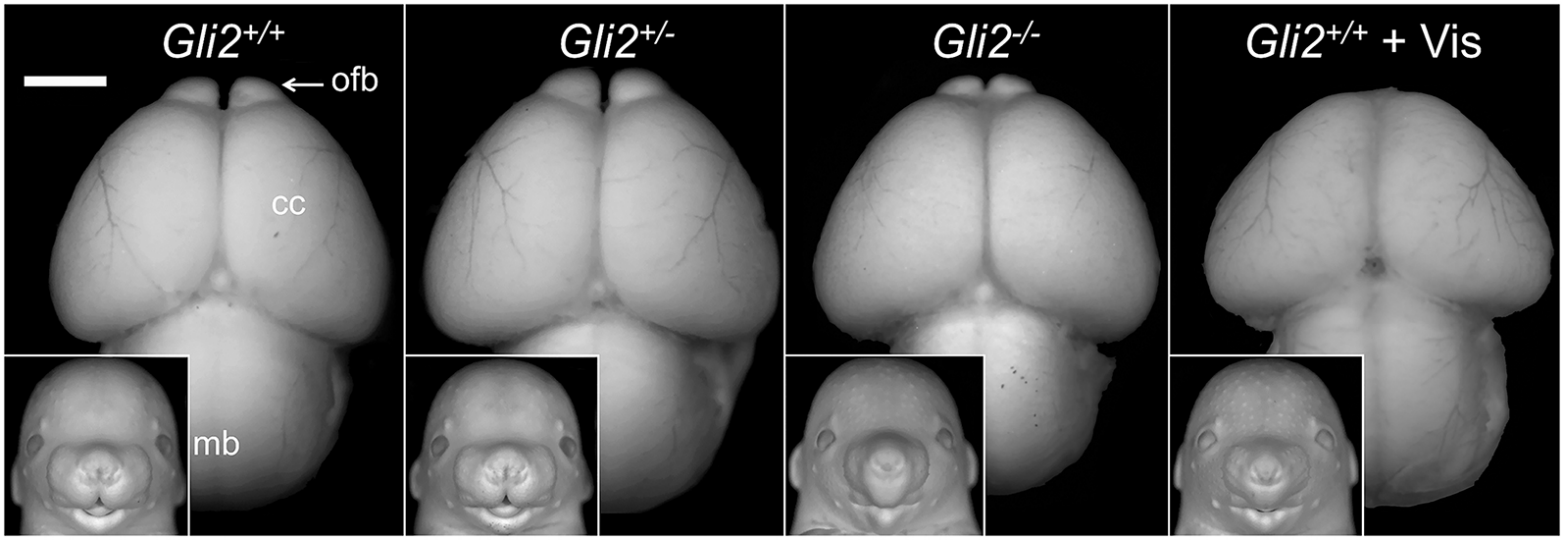
**Facial dysmorphology in *Gli2*^−/−^ fetuses co-occurs with brain abnormalities.** (A-D) Animals from the same experimental groups that are described in Fig. 1 are shown. To visualize correlative phenotypes, images of the dorsal surface of the brain along with facial images (inset) of the same animal are shown. Images were captured following Bouin's fixation and have been converted to grayscale. In the *Gli2*^−/−^ brain, hypoplasia of the cerebral cortices (cc) and midbrain (mb) was apparent. The olfactory bulbs (ofb) were hypoplastic and abnormally approximated. In the *Gli2^+/+^* fetus that had been exposed to vismodegib, more severe deficiency of the cerebral cortices and absence of the olfactory bulbs was observed. Scale bar: 1 mm.

We next investigated whether facial dysmorphology resulting from GLI2 loss of function co-occurs with forebrain abnormalities. *Gli2^−/−^* fetuses exhibited deficiency of the midbrain and forebrain (Fig. 2). Narrowing of the anterior aspect of the cerebral cortices extended to the olfactory bulbs, which were hypoplastic and abnormally closely spaced. A ventral view of the forebrain further illustrated the medial deficiency observed in *Gli2*^−/−^ fetuses (Fig. S3)*.* Vismodegib exposure in wild-type animals resulted in a more pronounced deficiency of the cerebral cortices and olfactory bulb aplasia. Gross brain morphology was indistinguishable between *Gli2^+/−^* fetuses and their wild-type littermates.

**Fig. 3.**
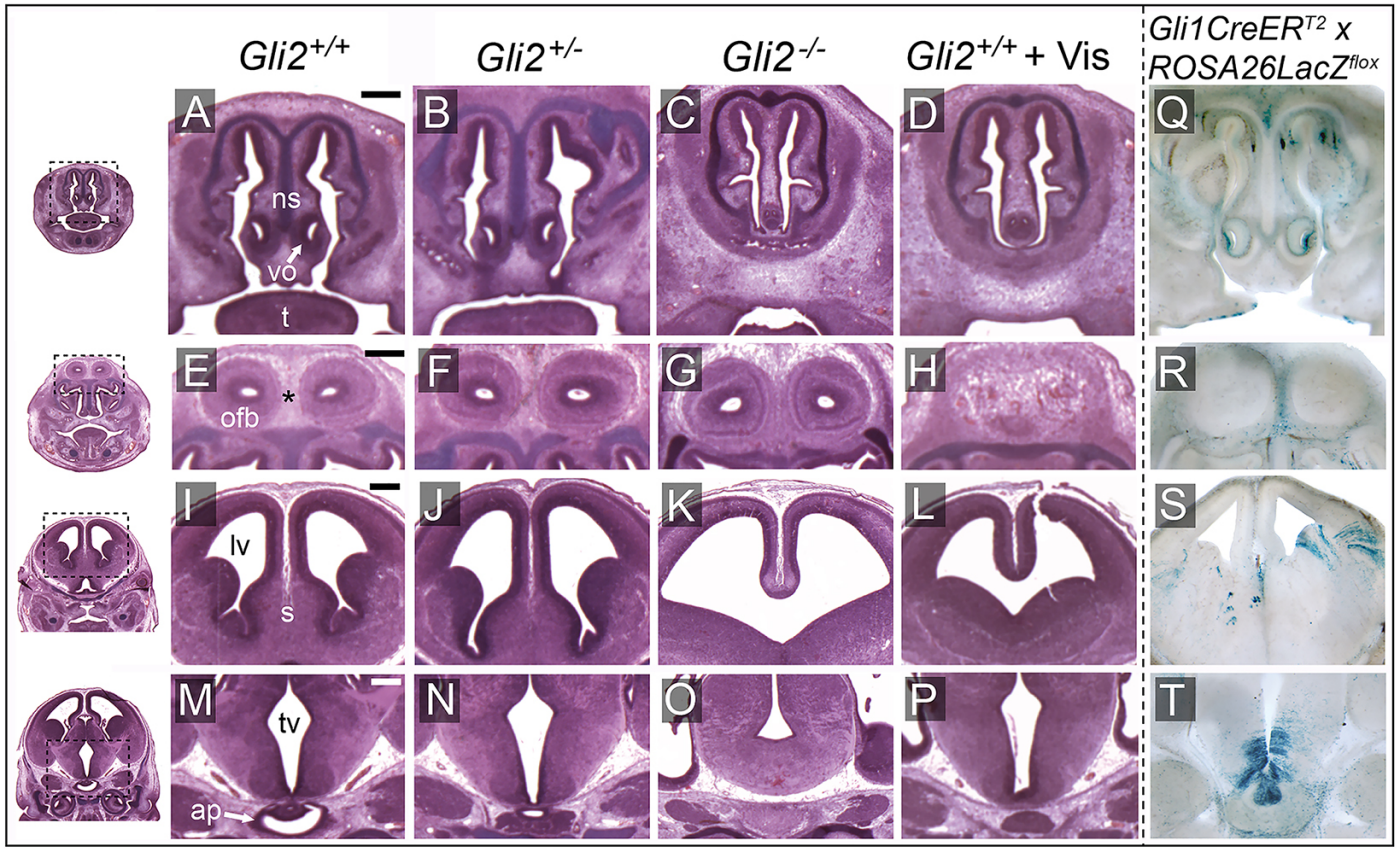
**GLI2 loss of function results in deficiency of medial forebrain and facial tissue.** (A-P) Serial histological (hematoxylin and eosin; H&E) images are shown from the same experimental groups described in Figs 1 and 2. Dashed boxes in the low-magnification images on the left provide context for the coronal sections shown in each row. *Gli2*^−/−^ and *Gli2^+/+^* vismodegib-exposed fetuses exhibited absence of nasal septal (ns) cartilage and diminished vomeronasal organs (vo). The midline connective tissue separating the olfactory bulbs (ofb) marked by * was reduced in area in the *Gi2*^−/−^ fetus, whereas the olfactory bulbs were absent in the *Gli2^+/+^* vismodegib-exposed fetus. These fetuses also had severe medial forebrain deficiencies, including absence of the septal region (s) and communicating lateral ventricles (lv). In the *Gli2*^−/−^ fetus, attenuation of the third ventricle (tv) and absence of the anterior pituitary (ap) lobe were also apparent. The anterior pituitary was present but hypoplastic in the *Gli2^+/+^* vismodegib-exposed fetus, which also exhibited subtle attenuation of the third ventricle. (Q-T) A genetic-fate mapping system (Ahn and Joyner, 2005) was used to identify Hh-responsive cell lineages. Embryos that had been exposed to tamoxifen at GD7.75 were sectioned and stained with X-Gal to visualize Hh-responsive cells and their progeny. 100-µM sections were produced at planes comparable to those that were stained with H&E. t, tongue. Scale bars: 0.25 mm.

The relationship between face and forebrain abnormalities resulting from GLI2 loss of function was investigated in more detail by performing histologic examination. This revealed specific features of midfacial hypoplasia, including absence of the nasal septal cartilage, closely spaced or fused nasal passages, and vomeronasal organ deficiency observed in each examined *Gli2^−/−^* fetus (*n*=6 out of 6) (Fig. 3C). One *Gli2^−/−^* fetus exhibited olfactory bulb agenesis, whereas, in each of the others (*n*=5 out of 6), the olfactory bulbs were present but spaced abnormally close together (Fig. 3G). Medial forebrain deficiency, evidenced by absence of the septal region, was observed in each B6 *Gli2^−/−^* fetus that was examined. The majority (*n*=4 out of 6) exhibited incomplete division of the cerebral cortices with a single communicating ventricle, the defining feature of HPE (Fig. 3K). Severe abnormalities in the diencephalic region of the forebrain were also observed. In all *Gli2^−/−^* fetuses, dysmorphology of the hypothalamus included attenuation of the development of the third ventricle, along with absence of both the anterior and posterior lobes of the pituitary (Fig. 3O). The brains and faces of *Gli2^+/−^* fetuses were histologically similar to those of their wild-type littermates.

The face and brain phenotypes observed in *Gli2^−/−^* fetuses were largely recapitulated by vismodegib exposure at GD7.75. Teratogen-exposed B6 wild-type mice exhibited absence of the nasal septal cartilage and forebrain septal region, and incomplete division of the cerebral cortices (Fig. 3D,L). Notable differences detected in vismodegib-exposed fetuses included higher penetrance of olfactory bulb agenesis, subtler diencephalic dysmorphology and a hypoplastic anterior pituitary (Fig. 3H,P).

Both GLI2 and the protein target of vismodegib, Smoothened, are expressed in SHH-ligand-responding cells, where they are required for transduction of the downstream signaling cascade (Lipinski et al., 2006; Robarge et al., 2009). Given the overlap in face and brain phenotypes between *Gli2*^−/−^ and vismodegib-exposed wild-type embryos, we hypothesized that tissue deficiency in these animals involves populations of cells that respond to SHH signaling at or shortly after GD7.75. To test this hypothesis, we used a tamoxifen-inducible lineage reporter model to trace temporally-specific Hh-responsive cells (Ahn and Joyner, 2005). Tamoxifen administration at GD7.75 revealed Hh-responsive cell lineages overlapping with cell populations found to be deficient in *Gli2*^−/−^ mice (Fig. 3Q-T). Regions with positive staining included vomeronasal organs, connective tissue between the olfactory bulbs, cerebral cortices, hypothalamus and anterior pituitary gland, indicating that these areas contain the progeny of cells that are responsive to Hh signaling at or shortly after GD7.75. This premise is further supported by the observation that expression of the conserved Hh-pathway target gene *Gli1* was reduced in the anterior neural plate and folds as early as GD8.0 in both *Gli2^−/−^* and vismodegib-exposed embryos (Fig. S4).

**Fig. 4.**
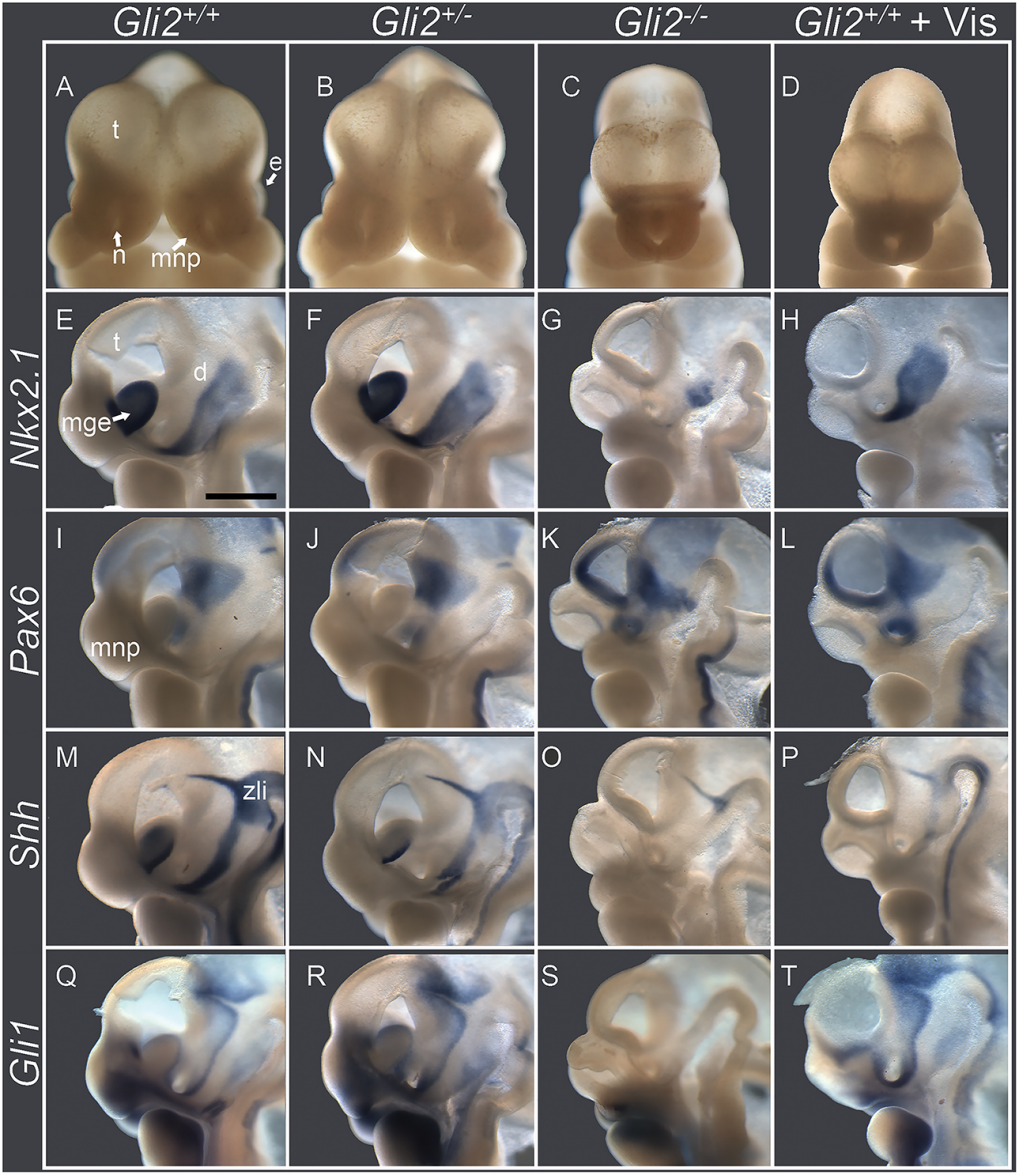
**GLI2 loss of function results in altered dorsal-ventral forebrain patterning.** Shown are embryos at GD11 of the same experimental groups described in Figs 1-3. (A-D) Frontal images show forebrain and facial morphology. *Gli2*^−/−^ and *Gli2^+/+^* fetuses that had been exposed to vismodegib exhibited hypoplasia and abnormal approximation of the telencephalic vesicles (t), absence of the medial nasal processes (mnp) and a single central nostril (n). (E-T) Embryos were hemisected near the midline and subjected to *in situ* hybridization for the indicated genes. In the *Gli2^+/+^* control, *Nkx2.1* was expressed in the medial ganglionic eminences (mge) and the ventral aspect of the diencephalon (d), whereas *Pax6* was present in the dorsal aspect of the telencephalon and diencephalon. *Shh* was expressed in the mantle region of the medial ganglionic eminence, along the ventral aspect of the diencephalon, and in the zona limitans intrathalamica (zli). *Gli1* expression reflects its paracrine responsiveness to stimulation with secreted SHH. e, eye. Scale bar: 1 mm.

Hh signaling plays two distinct roles in early forebrain development. It acts as a mitogen, promoting expansion of medial forebrain tissue and separation of the initially singular eye field. At the same time, SHH ligand secretion from the notochord and floor plate of the neural plate and tube specifies ventral neuroprogenitor cells. We tested whether attenuated forebrain expansion caused by GLI2 loss of function coincides with disruptions in early dorsal-ventral forebrain specification. At GD11.0, the morphogenesis of HPE was clearly evident in *Gli2^−/−^* embryos (Fig. 4C). This was highlighted by incomplete separation and hypoplasia of the telencephalic vesicles, along with absence of the medial nasal processes that form the median aspect of the upper lip and nose. As shown in a wild-type control that was hemisected in the sagittal plane, *Nkx2.1* is normally expressed in the medial ganglionic eminences and the ventral aspect of the diencephalon, whereas *Pax6* is present in dorsal aspects of the telencephalon and diencephalon (Corbin et al., 2003). *Gli2*^−/−^ embryos exhibited agenesis of the medial ganglionic eminences and absence of *Nkx2.1* expression in the telencephalon (Fig. 4G). Concurrently, the expression domain of *Pax6* was expanded into the ventral telencephalon (Fig. 4K). The altered forebrain patterning observed in *Gli2^−/−^* embryos was largely recapitulated through vismodegib exposure. However, the diencephalic domain of *Nkx2.1* expression was drastically reduced in *Gli2*^−/−^ embryos compared to those exposed to vismodegib. The expression domains of *Nkx2.1* and *Pax6* were indistinguishable between *Gli2*^+/−^ and *Gli2*^+/+^ embryos.

We also examined expression of *Shh* and *Gli1*, with the latter serving as a reliable indicator of Hh pathway activity. At GD11, *Shh* was normally expressed in the mantle region of the medial ganglionic eminences, along the ventral aspect of the diencephalon, and in the zona limitans intrathalamica (Fig. 4M). *Gli1* expression reflects its paracrine responsiveness to stimulation by secreted SHH. In *Gli2*^−/−^ embryos, expression of *Shh* and *Gli1* was nearly absent (Fig. 4O,S). In vismodegib-exposed wild-type embryos, *Shh* and *Gli1* expression was diminished but detectable in the ventral diencephalon and zona limitans intrathalamica (Fig. 4P,T).

### Gli2 heterozygosity increases sensitivity to teratogen-induced HPE

**Fig. 5.**
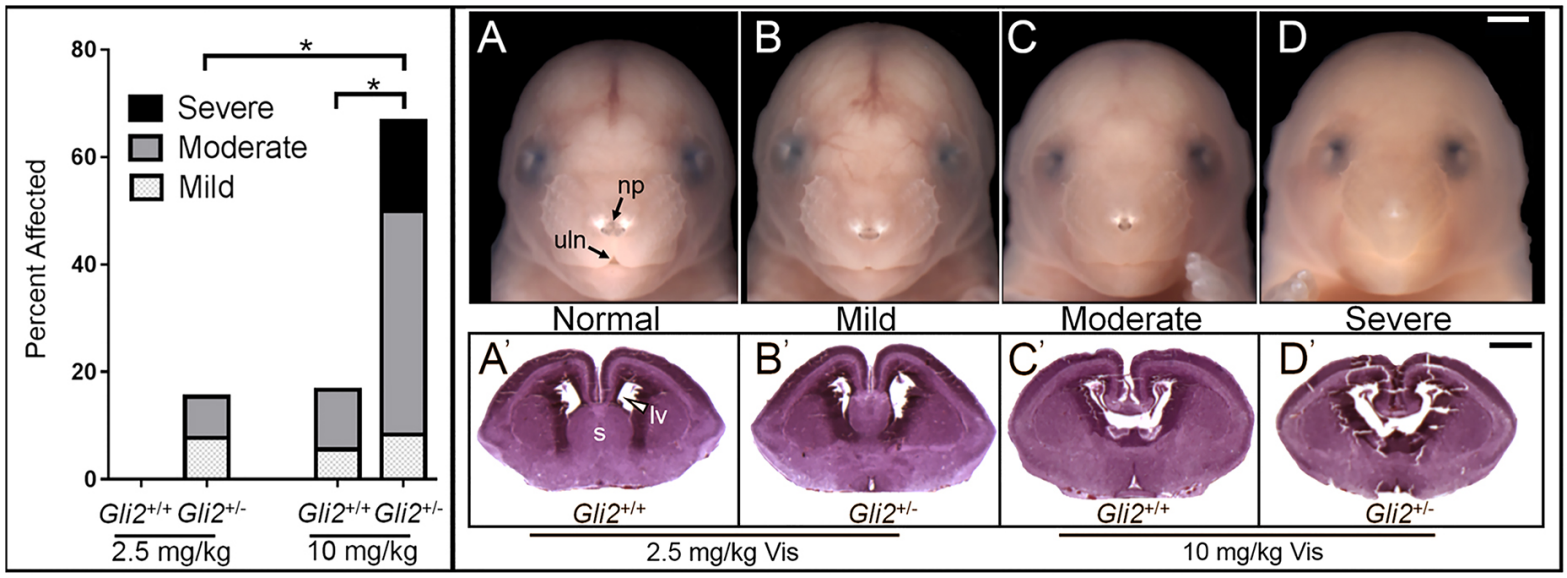
***Gli2* heterozygosity increases teratogenic sensitivity.** Wild-type dams carrying *Gli2^+/+^* and *Gli2^+/−^* embryos were exposed to 2.5 or 10 mg kg^−1^ vismodegib at GD7.75. (A-D) Categories of facial morphology are illustrated by GD17 fetuses of the indicated genotype and dose group. Animals that were indistinguishable from controls were assigned ‘normal’ (A). Those with a diminished area of pigmentation between the nostrils were assigned as ‘mild’ (B). Animals with a gross deficiency of the median lip notch but some remaining nasal pigment (np) at the tip of the nose were assigned as ‘moderate’ (C). Those with an absent lip notch (uln) and single central nostril were assigned as ‘severe’ (D). The graph on the left shows the percentage of GD17 fetuses that were classified as having mild, moderate or severe HPE-associated facial dysmorphology. *P<0.05 by Fisher's exact test. *n*=5 litters per treatment group. Data are mean±s.e.m. (A′-D′) Histologic sections through the cerebral cortices illustrate that the degree of facial dysmorphology corresponded to forebrain abnormalities. Note deficiency of the septal region (s) in B′ and the absent septal region and single communicating lateral ventricle (lv) in C′ and D′. Scale bars: 1 mm.

In surviving human cohorts with HPE-associated phenotypes, variants in the *GLI2* gene have been identified as heterozygous loss-of-function mutations (Roessler et al., 2003). Although severe phenotypes have been observed in some *GLI2*-mutation carriers, most exhibit incomplete HPE expressivity or are clinically normal. The data described above show that mice with single-allele *Gli2* mutations on the C57BL/6J background are phenotypically indistinguishable from their wild-type littermates. These observations support the argument that GLI2 is haplosufficient in the absence of additional genetic or environmental influences. To test whether normally silent single-allele *Gli2* mutations increase teratogenic sensitivity, direct comparison was made between wild-type and heterozygous littermates by mating *Gli2^+/+^* female and *Gli2^+/−^* male mice. As opposed to a 40 mg kg^−1^ dose utilized in the experiments described above, pregnant mice were exposed to a single dose of 2.5 or 10 mg kg^−1^ vismodegib at GD7.75. In the 2.5 mg kg^−1^ vismodegib exposure group, HPE-associated facial dysmorphology was detected in 15% (*n*=2 out of 13) of *Gli2^+/−^* fetuses but in none (*n*=0 out of 16) of the wild-type littermates (Fig. 5). In the 10 mg/kg vismodegib exposure group, dysmorphology was detected in 67% (*n*=8 out of 12) of *Gli2^+/−^* fetuses versus 17% (*n*=3 out of 18) of wild-type fetuses. The severity of vismodegib-induced facial dysmorphology was also dramatically increased in *Gli2* heterozygous fetuses compared their wild-type littermates. Histologic examination confirmed that the degree of observed facial dysmorphology closely corresponded with the severity of forebrain abnormalities.

**Fig. 6.**
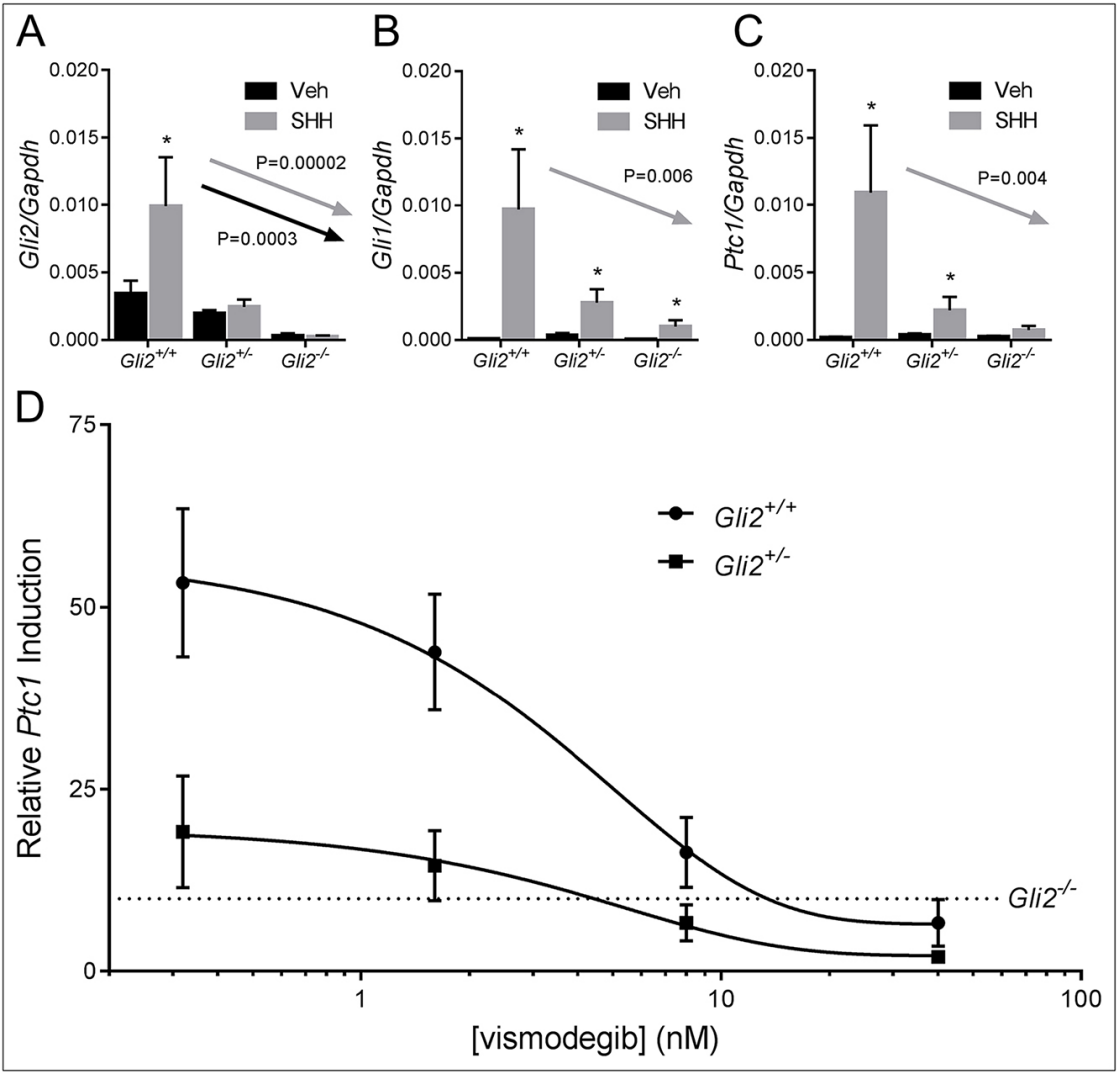
***Gli2* heterozygosity diminishes** **responsiveness to SHH ligand.** (A-C) *Gli2^+/+^*, *Gli2^+/−^* and *Gli2*^−/−^ mouse embryonic fibroblasts (MEFs) were treated with or without SHH ligand for 48 h. Expression of *Gli2* and the conserved Hh target genes *Gli1* and *Ptc1* were measured by performing real-time reverse transcriptase PCR, and expression was normalized to that of *Gapdh.* *P<0.05 as determined by Student's *t*-test for genotype-specific gene expression. Arrows indicate a significant gene-dosage effect, as determined by using a Jonckheere–Terpstra test. Veh, vehicle, DMSO. (D) *Gli2^+/+^* and *Gli2^+/−^* fibroblasts were treated with or without SHH ligand and with graded concentrations of vismodegib for 48 h. Induction of *Ptc1* (SHH relative to vehicle) relative to *Gli2^+/+^* cells is shown in the semi-log plot. The dashed line shows *Ptc1* induction (SHH relative to vehicle) in *Gli2*^−/−^ fibroblasts to illustrate a theoretical threshold of teratogenicity. For each graph, values represent the mean±s.e.m. of six replicate experiments.

We next investigated the effect of *Gli2* gene dosage on cellular responsiveness to SHH ligand using embryonic fibroblasts from B6 *Gli2^+/+^*, *Gli2^+/−^* and *Gli2^−/−^* mice. As expected, *Gli2* mRNA abundance was dependent upon the number of functional *Gli2* alleles (Fig. 6A). In *Gli2^+/+^* cells, stimulation with SHH peptide resulted in significant upregulation of the Hh target genes *Gli1* and *Ptc1*. Comparison of expression levels in *Gli2^+/+^*, *Gli2^+/−^* and *Gli2^−/−^* cells revealed a significant gene dosage effect. Specifically, the SHH-induced expression of these genes incrementally decreased with loss of each functional *Gli2* allele (Fig. 6B,C). We then examined the effect of *Gli2* haploinsufficiency on pathway sensitivity to vismodegib. *Gli2^+/+^* and *Gli2^+/−^* cells appeared to be equally responsive to pathway inhibition by vismodegib. However, because of diminished capacity to respond to SHH stimulation, target gene expression was lower in *Gli2^+/−^* cells at all vismodegib concentrations (Fig. 6D). Relative to *Gli2*^+/+^ cells, *Gli2^+/−^* cells reached the level of gene expression observed in *Gli2^−/−^* cells at a lower concentration of vismodegib*.* This *in vitro* result is consistent with the increased teratogenic sensitivity observed in *Gli2* heterozygous mice.

## DISCUSSION

Considerable effort has been made to elucidate the complex etiology of HPE, with recent studies focusing on underlying genetic factors. However, most identified mutations are heterozygous and demonstrate incomplete penetrance or expressivity. The tenuous connection between *GLI2* and HPE had typified this paradigm*.* Although the defining medial forebrain deficiency of true HPE has been identified in several individuals with single-allele *GLI2* mutations, most cases involve more subtle abnormalities, such as pituitary deficiency and/or facial dysmorphology (Bear et al., 2014; Roessler et al., 2003; Bertolacini et al., 2012; Franca et al., 2010; Rahimov et al., 2006). One explanation, proposed by Roessler et al., is that HPE results from additional environmental and genetic influences superimposed on the GLI2 haploinsufficient state (Roessler et al., 2003). This has become a widely accepted overarching premise of HPE etiology, but although some interacting factors have been identified, the majority of cases cannot be explained (Kietzman et al., 2014; Hong and Krauss, 2012; Roessler and Muenke, 2010).

The studies described herein directly address the complex etiology of HPE and provide a framework to understand the extreme phenotypic variability observed in human *GLI2-*mutation carriers. We provide the first evidence definitively linking GLI2 to HPE by demonstrating that a homozygous mutation causes the defining brain and face malformations. We also show that a single-allele mutation is normally silent but dramatically increases sensitivity to HPE that is induced by low-dose teratogen exposure. These findings establish a novel model to further elucidate the intricate gene-environment interactions that have frustrated clinical management of this common human birth defect.

In addition to variable clinical findings, the uncertain role of GLI2 in HPE pathogenesis was obfuscated by the initial characterization of *Gli2*-knockout mice. These mice were generated and maintained on an outbred CD-1/129 background and are described to have incompletely penetrant abnormalities of the vertebrae and limbs, as well as secondary palate clefts, pituitary hypoplasia and midbrain deficiency (Mo et al., 1997; Matise et al., 1998; Park et al., 2000). However, the forebrain and midfacial deficiency phenotypes characteristic of HPE are not observed in such animals (Blaess et al., 2006). This discordance extended to cellular and molecular findings. *Gli2^−/−^* fetuses on an outbred CD-1 background have intact expression of the ventral neurospecification marker *Nkx2.1* and grossly normal medial ganglionic eminences (Park et al., 2000). These transient developmental structures give rise to inhibitory cortical interneurons – cell populations that are depleted in individuals with true HPE (Fertuzinhos et al., 2009). Here, we demonstrate that when backcrossed to the C57BL/6J background, homozygous *Gli2* mutations cause the characteristic brain and face abnormalities associated with severe HPE, including severe diminishment of the medial ganglionic eminences, medial forebrain deficiency, hypotelorism and midfacial hypoplasia. These structural malformations followed diminished Hh-signaling pathway activity and severely disrupted forebrain patterning.

Backcrossing gene mutations to the C57BL/6 background has previously been shown to reveal or exacerbate HPE penetrance and/or expressivity. Specific examples include homozygous mutations in the Hh-ligand co-receptor *Cdo*, a predicted truncating mutation in *Tgif* and single-allele mutations in *Six3*, which is a transcription factor upstream of *Shh* (Kuang et al., 2006; Zhang et al., 2006; Geng et al., 2008). These reports, along with the findings presented in this study, suggest that the C57BL/6 background includes one or more yet-to-be-identified HPE modifier genes. Demonstration that GLI2 loss of function on the B6 inbred strain results in severe HPE phenotypes with complete phenotypic penetrance provides a new opportunity to uncover background-specific interacting genetic variations that could represent previously unidentified and clinically relevant predisposing factors.

Although this study has focused on gene-environment interaction, gene-gene interactions have been postulated to play a role in the complex etiology of HPE. As a basic test of this concept, we generated mice heterozygous for *Gli2* and *Shh*, the latter being the most commonly mutated gene identified in non-chromosomal HPE. When backcrossed to the C57BL/6J background, *Shh^−/−^* embryos exhibited severe HPE phenotypes, including severe midfacial hypoplasia and a proboscis (Fig. S5). However, *Shh^+/−^Gli2^+/−^* fetuses were apparently normal and indistinguishable from single heterozygotes and wild-type littermates. This suggests that *Shh* heterozygosity does not interact with *Gli2* haploinsufficiency. A functional interaction with a downstream gene is perhaps more likely given the role of GLI2 as a direct activator of Hh target genes. However, the tissue-specific Hh target genes that are regulated by GLI2 and mediate the pathogenesis of HPE are unknown. Moving forward, use of the complementary genetic and teratogenic models of HPE characterized here provide ideal platforms to identify Hh target genes involved in HPE pathogenesis and to identify gene-gene interactions.

GLI2 loss of function causes chronic Hh pathway disruption; however, the effect of acute pathway disruption can be examined by targeted exposure to the potent and specific inhibitor vismodegib. We found that the anatomical and molecular phenotypes caused by GLI2 loss of function were largely recapitulated by acute exposure to vismodegib at GD7.75. This observation suggests that the abnormalities we observed in GD15 fetuses are caused in part by changes to cell populations that respond to SHH signaling during the early neurulation stage of embryogenesis. This period of development (GD8.0 and shortly after) corresponds to the fourth week of human gestation and has been shown to be sensitive to other chemicals that cause HPE (Lipinski et al., 2010). This premise was further supported by genetic-fate mapping using an inducible system that labels Hh-responsive cells and their progeny at discrete periods of development. Tamoxifen exposure at GD7.75 revealed Hh-responsive cell lineages in specific medial facial and forebrain compartments, which corresponded to tissues that were deficient in *Gli2^−/−^* and vismodegib-exposed mice. However, several differences were also observed between the genetic and teratogenic models of HPE that we investigated. *Gli2^−/−^* animals exhibited more severe pituitary and diencephalic abnormalities but subtler olfactory bulb deficiency than those exposed to vismodegib. Loss of GLI2 function also resulted in a more extensive disruption of Hh-pathway activity and ventral specification in the diencephalic region of the forebrain. These differences are likely to reflect, at least in part, the pathologic effects of chronic versus acute attenuation of the Hh pathway inherent in these models. Additional factors contributing to these differences, like differential effects on Gli3 repression, are also possible and should be considered in future investigations.

Directly addressing the premise of gene-environment interaction, our study demonstrates that normally silent single-allele *Gli2* mutations dramatically increase teratogenic sensitivity. This result is congruous with our previous study that examined the effect of prenatal alcohol exposure in the context of *Gli2* heterozygosity (Kietzman et al., 2014). These findings illustrate that a functional predisposition can exacerbate the teratogenic effects of two unique classes of environmental influences, resulting in severe birth defects with largely overlapping phenotypes. Taken together, these findings provide a construct for understanding the extremely variable phenotypes that are exhibited in clinical populations and highlight the emerging consensus that HPE and other birth defects are likely to result from complex interactions between genetic and environmental factors.

We utilized the potent Hh-pathway antagonist vismodegib to test the concept of a gene-environment interaction in the context of *Gli2* heterozygosity. Importantly, vismodegib is part of a larger class of compounds that inhibit Hh signaling by binding to the transmembrane protein Smoothened (Robarge et al., 2009; Chen et al., 2002; Keeler, 1975; Chen, 2016). With both synthetic and natural small molecules, this group includes more than 20 compounds and continues to grow, with increasing attention focused on the biological effects of Hh-pathway inhibition. With respect to HPE etiology, perhaps the most intriguing Hh-pathway inhibitors are known environmental small molecules, including dietary alkaloids, an antifungal agent, a human dietary supplement and a pesticide synergist that is present in hundreds of insecticide formulations (Lipinski et al., 2007; Lipinski and Bushman, 2010; Wang et al., 2012). These compounds are less potent than vismodegib and appear unlikely to act independently to cause birth defects at typical environmental concentrations. However, the findings presented here illustrate the ‘multiple hit’ hypothesis of complex disease and should prompt careful investigation of the etiological role of environmental Hh-pathway inhibitors in the context of predisposing mutations in genes such as *Gli2*. Building upon the study presented here, continued elucidation of the intricate gene-environment interactions that cause HPE provides a direct path to improving clinical management and developing effective prevention strategies.

## MATERIALS AND METHODS

### Animals models

Mouse (*Mus musculus*) studies were performed in strict accordance with the recommendations in the Guide for the Care and Use of Laboratory Animals of the National Institutes of Health. The protocol was approved by the University of Wisconsin School of Veterinary Medicine Institutional Animal Care and Use Committee (protocol number 13-081.0). *Gli2^+/−^* mice (Matise et al., 1998) were backcrossed to the C57BL/6J background for more than 15 generations. C57BL/6J wild-type mice were purchased from The Jackson Laboratory (Bar Harbor, ME). *Gli1CreER^T2^* (stock no: 007913) and Rosa26lacZ (stock no: 003474) mice were purchased from The Jackson Laboratory. All mice were housed under specific pathogen-free conditions in disposable ventilated cages (Innovive, San Diego, CA) in rooms maintained at 22±2°C and 30-70% humidity on a 12-h light: 12-h dark cycle. Mice were fed 1919× Irradiated Harlan Teklad Global Soy Protein-Free Extruded Rodent Diet. For timed matings, 1-3 female mice between 8 and 20 weeks of age were placed with a single male for 1-2 h and subsequently examined for the presence of copulation plugs. The beginning of the mating period was designated as GD0. True pregnancy was confirmed by assessing weight gain between GD7 and 10 before dam treatment or embryo harvest as previously described (Heyne et al., 2015b).

### Hh-signaling antagonist exposure

Vismodegib was purchased from LC Laboratories (Woburn, MA) and suspended at 3 mg ml^−1^ in 0.5% methyl cellulose with 0.2% Tween 80 as previously described (Heyne et al., 2015a). Individual suspensions were prepared within 30 min of administration. Pregnant mice were administered 2.5, 10 or 40 mg kg^−1^ vismodegib (aka GDC-0449) by oral gavage at GD7.75.

### Dissection, imaging and fetal phenotyping

Pregnant dams were euthanized at indicated stages of development by CO_2_ asphyxiation and subsequent cervical dislocation. Fetal specimens were fixed in 10% formalin or Bouin's solution for at least 1 week before imaging. Images were captured using a micropublisher 5.0 camera connected to a Nikon SZX-10 stereomicroscope. Linear facial measurements of formalin-fixed fetuses at GD15 were produced in Photoshop v14.1.2 as previously described (Lipinski et al., 2014). For comparison of face and brain morphology, images of Bouin's-fixed tissue were captured and converted to grayscale. The severity of HPE phenotypes in GD17 fetuses was assessed by a single rater that had been blinded to treatment based upon a semi-quantitative scale as previously described (Heyne et al., 2015a; Kietzman et al., 2014).

### Histology

GD15 fetuses were fixed in Bouin's solution for at least 1 week and then transferred to 70% ethanol. GD17 fetuses were fixed in 10% formalin. Following paraffin embedding, 10-μm sections were produced and stained with H&E by standard protocols.

### Fate mapping

*Gli1CreER^T2+/−^* male mice were mated with *Rosa26lacZ^flox/flox^* females. 50 mg ml^−1^ tamoxifen that had been dissolved in corn oil was administered to pregnant dams through intraperitoneal injection at GD7.75. GD15 fetuses were fixed overnight in 2% paraformaldehyde with 0.2% glutaraldehyde. Embryonic tissues were then embedded in 4% agarose and 150-μm coronal sections were produced by using a vibrating microtome and were then X-Gal-stained as described previously (Mehta et al., 2011).

### *In situ* hybridization

*In situ* hybridization was performed using an established high-throughput technique that allows multiple treatment groups to be processed identically and as a single unit (Abler et al., 2011). For GD11 embryos, mid-sagittal hemisection using a scalpel was performed before staining. Hemisected embryos were incubated in proteinase K (2.5 µg ml^−1^) with collagenase (250 µg ml^−1^) for 2 min before initial washes. Younger embryos were not subjected to proteinase K or collagenase treatment. *In situ* hybridization probe primers were resuspended as stock solutions of 100 mM in Tris-EDTA buffer pH 7.0 (Ambion). Working stocks were made as 10 mM solutions containing both forward and reverse primers. The primer sequences used for probe generation are listed in Table S1.

### Mouse embryonic fibroblast isolation and treatment

Mouse embryonic fibroblasts were harvested from GD15 embryos as previously described (Lipinski et al., 2006). Cells were grown to confluence in Dulbecco's modified Eagle's medium (DMEM) (with l-glutamine, 4.5 g l^−1^ glucose without sodium pyruvate) with 10% FCS and 1% penicillin-streptomycin. They were then trypsinized and plated in 24- or 48-well tissue culture plates (Falcon, Franklin Lakes, NJ) at 4.0×10^5^ cells ml^−1^ media. Cells were allowed to attach for 24 h, and media were replaced with DMEM containing 1% FBS with or without the active SHH N-terminal peptide (R&D Systems, Minneapolis, MN) at 0.4 μg ml^−1^, with or without vismodegib (LC Laboratories) at indicated concentrations. For cell culture experiments, vismodegib was dissolved in DMSO.

### RNA isolation and real-time reverse-transcriptase PCR

RNA was isolated using GE Illustra RNAspin kits. 100 ng of RNA was reverse transcribed using the Promega GoScript Reverse Transcription System. Both kits were used according to manufacturer's instructions. Real-time PCR was performed using a Bio-Rad CFX96 Touch real-time PCR detection system. Reaction mixtures contained 6 µl SSoFast EvaGreen Supermix (Bio-Rad Laboratories, Hercules, CA), 4.75 µl ddH_2_O, 0.75 µl cDNA and 0.5 µl 10 mM combined forward and reverse gene-specific primers. Primers were resuspended as stock solutions of 100 mM in Tris-EDTA buffer pH 7.0. Working stocks were made as 10 mM solutions containing both forward and reverse primers. Primer sequences are listed in Table S2. Reaction conditions were as follows: 1 cycle at 95°C for 3 min, then 40 cycles of 95°C for 10 s followed by 30 s at 60°C (annealing temperature). To confirm specificity, primer sequences were analyzed with BLAST and melt curves were examined for a single peak in the expected temperature range. *Gapdh* was used as the housekeeping gene, and analysis was conducted with the 2^ΔΔ^^Ct^ method.

### Statistics

Analysis of linear measurements was made using one-way ANOVA followed by Tukey's honest significant difference (HSD) test using Graphpad Prism software (v6.04). Differences in the frequency of facial dysmorphology between experimental groups were assessed by using one-tailed Fisher's exact test using Graphpad Prism software. Student's *t*-tests were used to determine whether gene expression was changed by SHH stimulation in mouse embryonic fibroblasts. To test whether basal and SHH-induced target gene expression preserved the ordering implicitly suggested by the genotypes, a Jonckheere–Terpstra test was performed using Mstat version 6.1.4 (http:/mcardle.oncology.wisc.edu/mstat/download/index.html). An α value of 0.05 was maintained for all analyses.

### Human images

Parental written informed consent was received for inclusion and publication of the image of the HPE-affected individual in Fig. 1.
